# SARS-CoV-2 Omicron Variants Show Attenuated Neurovirulence Compared with the Wild-Type Strain in Elderly Human Brain Spheroids

**DOI:** 10.34133/research.0376

**Published:** 2024-05-13

**Authors:** Weikang Chen, Xiaobing Jiang, Wei Liang, Haojie Bai, Mingze Xu, Zhe Liu, Lina Yi, Yanming Liu, Yanxia Huang, Yongxin Zhang, Lixia Xu, Baoshu Xie, Nu Zhang, Jun Yu, Jing Lu, Haipeng Xiao, Xiaoxing Li

**Affiliations:** ^1^ Department of Neurosurgery, The First Affiliated Hospital, Sun Yat-sen University, Guangzhou 510000, China.; ^2^ Institute of Precision Medicine, The First Affiliated Hospital, Sun Yat-sen University, Guangzhou 510000, China.; ^3^ Department of Neurosurgery/Neuro-Oncology, State Key Laboratory of Oncology in South China, Guangdong Provincial Clinical Research Center for Cancer, Sun Yat-sen University Cancer Center (SYSUCC), Guangzhou 510000, China.; ^4^ Guangdong Provincial Institution of Public Health, Guangdong Provincial Center for Disease Control and Prevention, Guangzhou 510000, China.; ^5^ Department of Oncology, The First Affiliated Hospital, Sun Yat-sen University, Guangzhou 510000, China.; ^6^ Department of Medicine and Therapeutics and Institute of Digestive Disease, State Key Laboratory of Digestive Disease, The Chinese University of Hong Kong, Hong Kong SAR 999077, China.; ^7^ Department of Endocrinology, The First Affiliated Hospital, Sun Yat-sen University, Guangzhou 510000, China.

## Abstract

Infection with severe acute respiratory syndrome coronavirus 2 Omicron variants still causes neurological complications in elderly individuals. However, whether and how aging brains are affected by Omicron variants in terms of neuroinvasiveness and neurovirulence are unknown. Here, we utilize resected paracarcinoma brain tissue from elderly individuals to generate primary brain spheroids (BSs) for investigating the replication capability of live wild-type (WT) strain and Omicron (BA.1/BA.2), as well as the mechanisms underlying their neurobiological effects. We find that both WT and Omicron BA.1/BA.2 are able to enter BSs but weakly replicate. There is no difference between Omicron BA.1/BA.2 and WT strains in neurotropism in aging BSs. However, Omicron BA.1/BA.2 exhibits ameliorating neurological damage. Transcriptional profiling indicates that Omicron BA.1/BA.2 induces a lower neuroinflammatory response than WT strain in elderly BSs, suggesting a mechanistic explanation for their attenuated neuropathogenicity. Moreover, we find that both Omicron BA.1/BA.2 and WT strain infections disrupt neural network activity associated with neurodegenerative disorders by causing neuron degeneration and amyloid-β deposition in elderly BSs. These results uncover Omicron-specific mechanisms and cellular immune responses associated with severe acute respiratory syndrome coronavirus 2-induced neurological complications.

## Introduction

Infection with severe acute respiratory syndrome coronavirus 2 (SARS-CoV-2) causes a wide range of neurological complications, including loss of smell, headaches, and encephalitis [1]. A substantial number of patients suffering from long COVID may persist as “brain fog”, characterized by difficulty paying attention, remembering, and processing information [2,3]. It is particularly concerning for older people (65 years or older) who are at higher risk of COVID-19 symptoms persisting than younger people [4,5]. In some patients with COVID-19 and concomitant neurological symptoms, cerebrospinal fluid and autopsy brain tissue have tested positive for SARS-CoV-2 viral RNA and protein [6,7]. Recent studies in rodents have suggested that direct viral invasion of neural tissue or secondary consequences of the immune response may contribute to the pathogenesis of SARS-CoV-2-associated central nervous system diseases [8–10]. To date, the nature of infection and the underlying mechanisms of neurological sequelae induced by SARS-CoV-2 in elderly brain tissue remain poorly understood.

During the global Omicron wave, several studies have indicated that older people are more likely to be affected by COVID-19 [11]. According to COVID-19 guidelines issued by the Centers for Disease Control and Prevention, patients aged 50 to 64 were 4.3 times as likely to progress to severe status as those aged 18 to 39, and patients aged 85 or older had a risk ratio of 10.6. During the Omicron wave in Shanghai, a study found that 48.1% of patients with COVID-19 (average age, 70.6 years) infected with the Omicron strain experienced neurological symptoms [12]. Recent studies have shown that Omicron sublineages have altered virological characteristics, including increased immune evasion and attenuated pathogenicity, compared to previous variants [13–15]. However, it is unclear whether and how Omicron variants have different neuroinvasiveness and neurovirulence in the elderly brain. Brain organoids generated from human pluripotent stem cells have been effectively utilized to investigate the neurotropism and neurotoxic consequences of SARS-CoV-2 [16–18]. However, stem-cell-derived brain organoids recapitulate the early stages of brain development and lack nonneuronal cell types, including microglia and endothelial cells [19], and older mice rapidly died following wild-type (WT) strain infection. Thus, stem cell model and older mouse model are not suitable for evaluating the differences of WT and Omicron in the aging brain.

In this study, we first generated brain spheroids (BSs) derived from paracarcinoma tissues from patients with brain tumors and then evaluated the differences in neuroinvasiveness and neurovirulence in Omicron variants (BA.1 and BA.2) compared to the WT strain using elderly BSs. We observed that Omicron variants (especially BA.2) have markedly lower neurovirulence. Omicron BA.1/BA.2 infection induces weaker neuroinflammation than the WT strain, which may explain its neuropathological changes. Moreover, Omicron BA.1/BA.2 and WT strain infections are associated with neurodegenerative disorders by triggering neuronal degeneration and amyloid-β (Aβ) deposition.

## Results

### Establishment of primary human brain tissue-derived elderly BSs

**Fig. 1.**
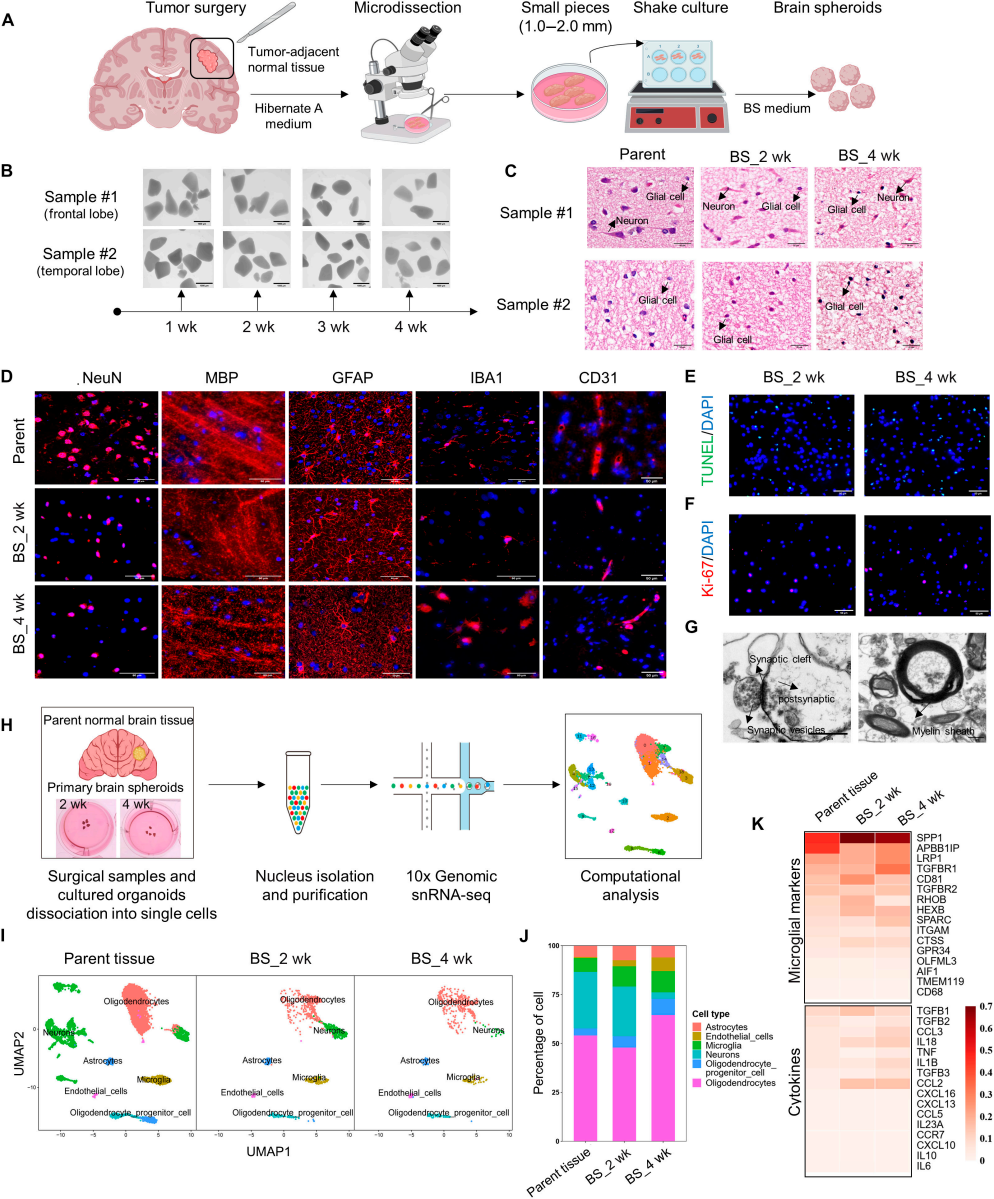
Generation of BSs that retain histologic features and cellular heterogeneity of parental tissue. (A) Schematic of the procedure for sample treatment and BS culture. (B) Timeline showing representative images of BS cultures. Scale bars, 1,000 μm. (C) H&E staining images of parental brain tissue and corresponding spheroids. Scale bars, 50 μm. (D) IFA staining for neuronal nuclei (NeuN), MBP, GFAP, IBA1, and CD31. Scale bars, 50 μm. (E and F) IFA staining for TUNEL and Ki-67 at cultured BSs. Scale bars, 50 μm. PFU, plaque-forming units. (G) Electron microscopy of characteristic synapses and myelin sheath in BSs. Scale bars, 1 μm. (H) Schematic for the generation of snRNA-seq data. Parental frontal lobe tissue and corresponding primary BSs from a 59-year-old patient. (I) UMAP plots of parental tissue and corresponding primary BSs. (J) The proportion of each cell type in parental tissue and corresponding primary BSs. (K) Heatmap of the gene expression of microglial marker genes and cytokines in the microglial cell cluster.

To model SARS-CoV-2 WT and Omicron infections on elderly human brain in vitro in physiologically relevant models, we established a new method to generate primary elderly BSs without mechanically or enzymatically dividing the removed paracarcinoma brain tissue into single cells (Fig. 1A) by referencing publications on generating glioblastoma organoids from patient tumors [20–22]. As shown in Table S1, we obtained fresh brain tissue dissected from 9 patient paracancerous samples (average age, 62 years; 6 temporal lobes and 3 frontal lobes). Optimal BSs were generated from surrounding brain tissue far from the tumor’s edge. To improve nutrient absorption and promote spheroid development, we cut the brain tissue into pieces around 1 mm in diameter and cultured it in BS medium on an orbital shaker (Fig. 1A). Fragments of brain tissue generally formed spheroids in 1 to 2 weeks, and the spheroids did not show marked growth but can be viably maintained during 1 months of culture without passaging (Fig. 1B).

To assess whether BSs are similar to the original parent tissue, we performed histological analysis of the parent tissue and spheroid sections by hematoxylin and eosin (H&E) and immunofluorescence assay (IFA) staining (Fig. 1C and D and Fig. S1A and B). We observed that the cultured BSs displayed the basic cellular composition of nervous tissue, maintained parental tissue neural marker expression, and also retained CD31^+^ vasculature. Small numbers of apoptotic and proliferative cells were observed in BSs, which indicated that the spheroids were viable and had no marked apoptosis (Fig. 1E and F). Meanwhile, transmission electron microscopy (TEM) imaging of a spheroid at 2 weeks confirmed the maintenance of structurally defined synapses and myelin sheath (Fig. 1G). Therefore, these data indicated that elderly BSs derived from patient paracarcinoma brain tissue would capture broad cellular diversity and complex organization of the original brain tissue.

### Preservation of cellular heterogeneity and molecular signatures of primary elderly BSs

Next, we compared the cell populations and their molecular characteristics of the original brain tissue and their derived BSs to further characterize the BS models. We performed single-nucleus RNA sequencing (snRNA-seq) analysis of frontal lobe tissue from a 59-year-old patient and corresponding BSs cultured 2 and 4 weeks (Fig. 1H). A median of 2,807 genes were found per cell after low-quality cells were eliminated, leaving 11,971 cells for biological study (Fig. S1C). The Uniform Manifold Approximation and Projection (UMAP) approach was used to analyze transcriptional profiles of individual nuclei in 2 dimensions. Global clustering of snRNA-seq revealed 19 major populations, as indicated by marker genes that were preferentially expressed in each cluster (Fig. S1D and E). By using marker genes, these clusters may be linked to 6 distinct cell lineages, which include neurons, astrocytes, oligodendrocytes, oligodendrocyte progenitor cells, microglia, and endothelial cells (Fig. S1F).

Many different cell clusters were identified in parental brain tissue and corresponding BSs at 2 and 4 weeks (Fig. 1I and Fig. S1G). BSs are derived from a portion of the parent brain tissue that is highly heterogeneous, and some cell subpopulations are inevitably lost. However, overall, at the single-cell level, the cells from both parental brain tissue and derived BSs exhibited similar cell lineages and clusters, which included neurons (~25%), astrocytes (~6%), oligodendrocytes (~55%), oligodendrocyte progenitor cells (~6%), microglia (~8%), and endothelial cells (Fig. 1J and Fig. S1H). The proportion of each cell lineage and cluster was similar in parental tissues and BSs (especially after 2 weeks of culture). It should be noted that the proportion and subpopulation of neurons in BS change over culture time due to spheroid are parts of the parent brain and reduced adult neurogenesis in the aging brain. Microglia in the parent tissue and brain organoids at 2 or 4 weeks show comparable expression of numerous immune-related genes, including cytokines like transforming growth factor–B (*TGFB*), interleukin-18 (*IL18*), and *IL1B*. This indicates that some aspects of the tissue microenvironment are retained within the brain organoids (Fig. 1K). These results imply that the cellular diversity and molecular characteristics of the original brain tissue are reflected in elderly brain organoids cultures.

### Elderly BSs are permissive to SARS-CoV-2 WT and Omicron BA.1/BA.2 infections

**Fig. 2.**
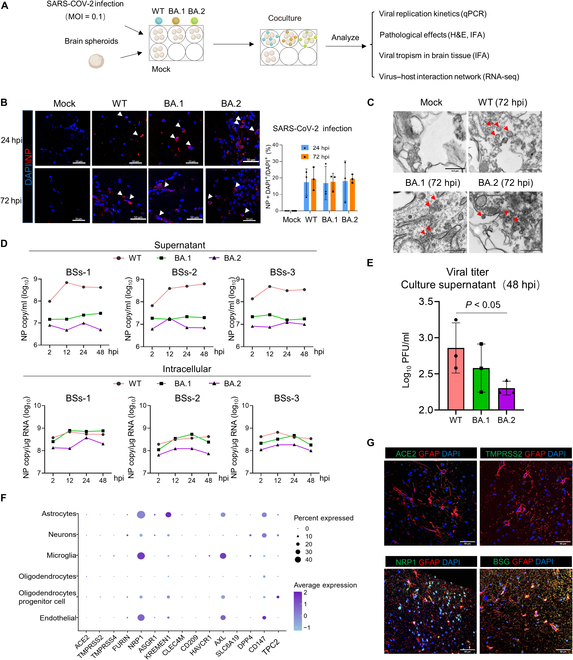
WT and Omicron BA.1/BA.2 live virus replication in elderly human BSs. (A) Experimental scheme for BSs and SARS-CoV-2 coculture establishment. (B) IFA staining of SARS-CoV-2-infected elderly BSs from the temporal lobe at an MOI of 0.1 at 24 and 72 hpi. Viral NP protein was stained red. Nuclei are shown with DAPI in blue. Scale bars, 50 μm. (C) Representative TEM images of elderly BSs from the temporal lobe at 72 h of WT and Omicron infections. Scale bars, 0.5 μm. (D) SARS-CoV-2 were inoculated into 3 elderly BSs at an MOI of 0.1, and the copy number of the viral RNA in the supernatant and cell lysates was quantified by qPCR. (E) Infectious viral particles were titrated using median tissue culture infectious dose assays. Statistical significance was determined with one-way ANOVA (F) Dot plot showing the average expression and percentage of cells expressing SARS-CoV-2 entry factors. (G) Expression of ACE2, TMPRSS2, NRP1, and CD147 in BSs.

To investigate the effects of Omicron infection on the elderly brain and its induced neuropathology (Fig. 2A), we exposed BSs from non-COVID-19 elderly individuals to live SARS-CoV-2 WT and Omicron BA.1/BA.2 at a multiplicity of infection (MOI) of 0.1 for 8 h. The samples were then analyzed at 24 and 72 h after infection (hpi). The presence of viral nucleocapsid protein (NP) was observed in both WT- and Omicron BA.1/BA.2-infected elderly BSs, with the percentage of infected cells ranging from 8.3% to 29.4%, but the number of infected cells in WT- and Omicron-infected BSs did not significantly increase from 24 to 72 hpi (Fig. 2B), suggesting limited spread of infection within this time frame. TEM examination identified typical coronavirus particles in WT- and Omicron-infected BSs from the temporal lobe at 72 hpi (Fig. 2C).

Furthermore, the replication kinetics of WT and Omicron BA.1/BA.2 were compared by examining viral titers in culture supernatants and BS lysates at 2, 12, 24, and 48 hpi (Fig. 2D). We detect an increase in viral RNA in the supernatants between 2 and 12 hpi in WT-infected BSs but insignificant increase in the supernatants of Omicron BA.1/BA.2-infected BSs. Meanwhile, slight increase in intracellular viral RNA was observed in all infection groups except the BA.2-infected BSs-1. Again, this trend was observed in BSs derived from 6 other individual brain samples (5 temporal lobes and 1 frontal lobe; average age, 64.8 years; range, 57 to 72 years). In these samples, the viral load of SARS-CoV-2 in the culture supernatant from WT-infected BSs at 72 hpi was 5.01 times higher (*P* = 0.032) and 9.33 times higher (*P* = 0.027) than that in BA.1- and BA.2-infected samples, respectively (Fig. S2A to C). The median tissue culture infectious dose assay also showed that the Omicron BA.1/BA.2 variant produced significantly less infectious virus particles at 48 hpi compared with SARS-CoV-2 WT (Fig. 2E). These results suggested that (a) elderly BSs were permissive to WT and Omicron strain infections, but both WT and Omicron BA.1/BA.2 do not actively replicate in BSs; (b) the brain organoids can only transiently (12 to 24 hpi) support the SARS-CoV-2 WT replication after the viral entry; (c) the increasing viral RNA can be only detected in WT-infected organoids highlighting the difference in virus release efficiency between WT and Omicron variants. It is possible that Omicron BA.1/BA.2 enters BSs but does not actively replicate by limiting the release of virus particles. There are several studies that have reported that SARS-CoV-2 is able to infect human induced-pluripotent-stem-cell-derived brain organoids/neurons but that this infection is abortive and does not result in virus spread to other cells [16,23].

**Fig. 3.**
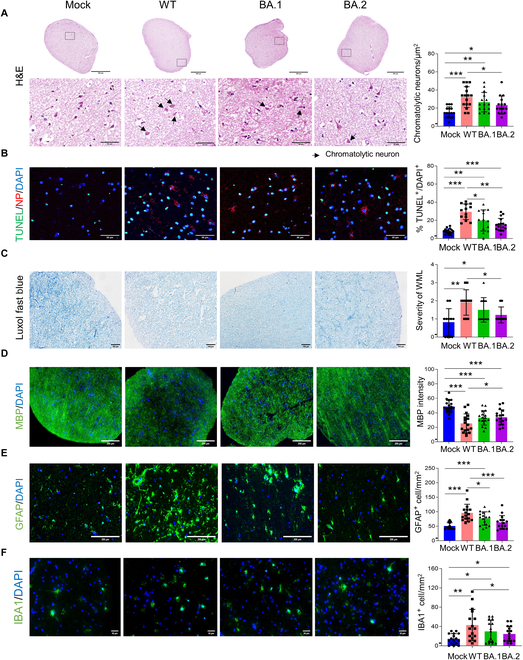
Neuropathological injury caused by WT and Omicron BA.1/BA.2 infections. (A) The neuronal nucleus shows reduced H&E staining and an increase in the size of the cell body and dispersed chromatin, while the cytoplasm appears paler and characterized by chromatolytic reactions (indicated by arrows). Scale bars, 50 μm. (B) IFA staining for TUNEL and viral NP protein in SARS-CoV-2-infected elderly BSs at 72 hpi. Nuclei are shown with DAPI in blue. Scale bars, 50 μm. (C) BSs infected with different SARS-CoV-2 strains were assayed for demyelination by Luxol fast blue staining, and the severity of white matter lesions (WML) was scored on a 0 to 3 scale. Scale bars, 100 μm. (D and E) Representative images of myelin content and astrogliosis in BS infection with SARS-CoV-2. Scale bars, 200 μm. (F) IFA staining for microglial marker (IBA1) in SARS-CoV-2-infected BSs. Nuclei are shown with DAPI in blue. Scale bars, 50 μm. Quantitative analysis of chromatolytic neurons (A), TUNEL-positive cells (B), demyelination (C), MBP, GFAP, and IBA1 expression (D to F) by counting 3 to 5 fields of the spheroids from the 4 individual elderly brain samples (3 temporal lobes and 1 frontal lobe). Data represent the means ± SD; statistical significance was determined with one-way ANOVA (A to F). *P < 0.05, **P < 0.01, and ***P < 0.001.

On the basis of the susceptibility of BSs to WT and Omicron BA.1/BA.2, the expression of known SARS-CoV-2 receptors was investigated. snRNA-seq analysis indicated that angiotensin-converting enzyme 2 (ACE2) and transmembrane protease, serine 2(TMPRSS2) mRNAs were almost undetected in all neural cell types; however, astrocytes, microglia, and endothelial cells expressed high mRNA levels of the alternative receptors neuropilin 1 (NRP1), CD147, and tyrosine-protein kinase receptor UFO (AXL) (Fig. 3F). IFA confirmed that BSs/astrocytes did not express ACE2 and TMPRSS2 but strongly expressed NRP1 and CD147 (Fig. 2G and Fig. S2D).

### SARS-CoV-2 Omicron BA.1/BA.2 infection shows ameliorated neuropathological injury compared to the WT strain

To further examine the impact of Omicron variant infection on neurological damage in BSs compared to the WT strain, formalin-fixed elderly BSs (3 temporal lobes and 1 frontal lobe) from SARS-CoV-2 infection at 72 hpi were analyzed for histology and immunohistochemistry evaluation. We found that scattered chromatolytic neurons were more likely to be found in BSs infected with WT than in those infected with Omicron BA.1/BA.2 (Fig. 3A). Chromatolysis is an induced response of the cell usually triggered by neurotoxicity, cell exhaustion, and virus infections, and it is a precursor of cell death [24]. We further evaluated apoptosis induction in the infected BSs by terminal-deoxynucleotidyl-transferase-mediated deoxyuridine triphosphate nick end labeling (TUNEL) immunostaining. In addition, both WT and Omicron strain infections induced significant apoptosis in BSs, but Omicron BA.1/BA.2 triggered lower apoptosis compared with that of WT strain (Fig. 3B).

Histology analysis of elderly BSs containing myelin sheath and Luxol fast blue staining showed that WT or Omicron BA.1 infection caused significant loss of myelin but attenuated myelin injury was observed in Omicron BA.2-infected BSs when compared with WT infection (Fig. 3C). In addition, staining for myelin basic protein (MBP) revealed substantial demyelination in BSs infected with the WT or Omicron BA.1/BA.2 variants, with less demyelination observed in those infected with the Omicron BA.2 variant compared to the WT strain (Fig. 3D). Furthermore, there was a notable increase in activated astrocytes (astrogliosis) in the areas of demyelination in BSs infected with the WT or Omicron BA.1 variants, but less astrogliosis was observed in those infected with the Omicron BA.1/BA.2 variants compared to the WT strain (Fig. 3E). IFA staining showed that there was a substantial increase in microglial (IBA1^+^ cells) number in WT- and BA.1/BA.2-infected BSs compared with mock-infected BSs, which was especially prominent in WT-infected elderly BSs (Fig. 3F). Overall, these findings suggest that both the WT and Omicron BA.1/BA.2 lead to neuropathological damage in the aging brain but the Omicron BA.1/BA.2 mitigate this damage to some extent.

### SARS-CoV-2 Omicron BA.1/BA.2 has no significant difference in neurotropism compared to the WT strain

**Fig. 4.**
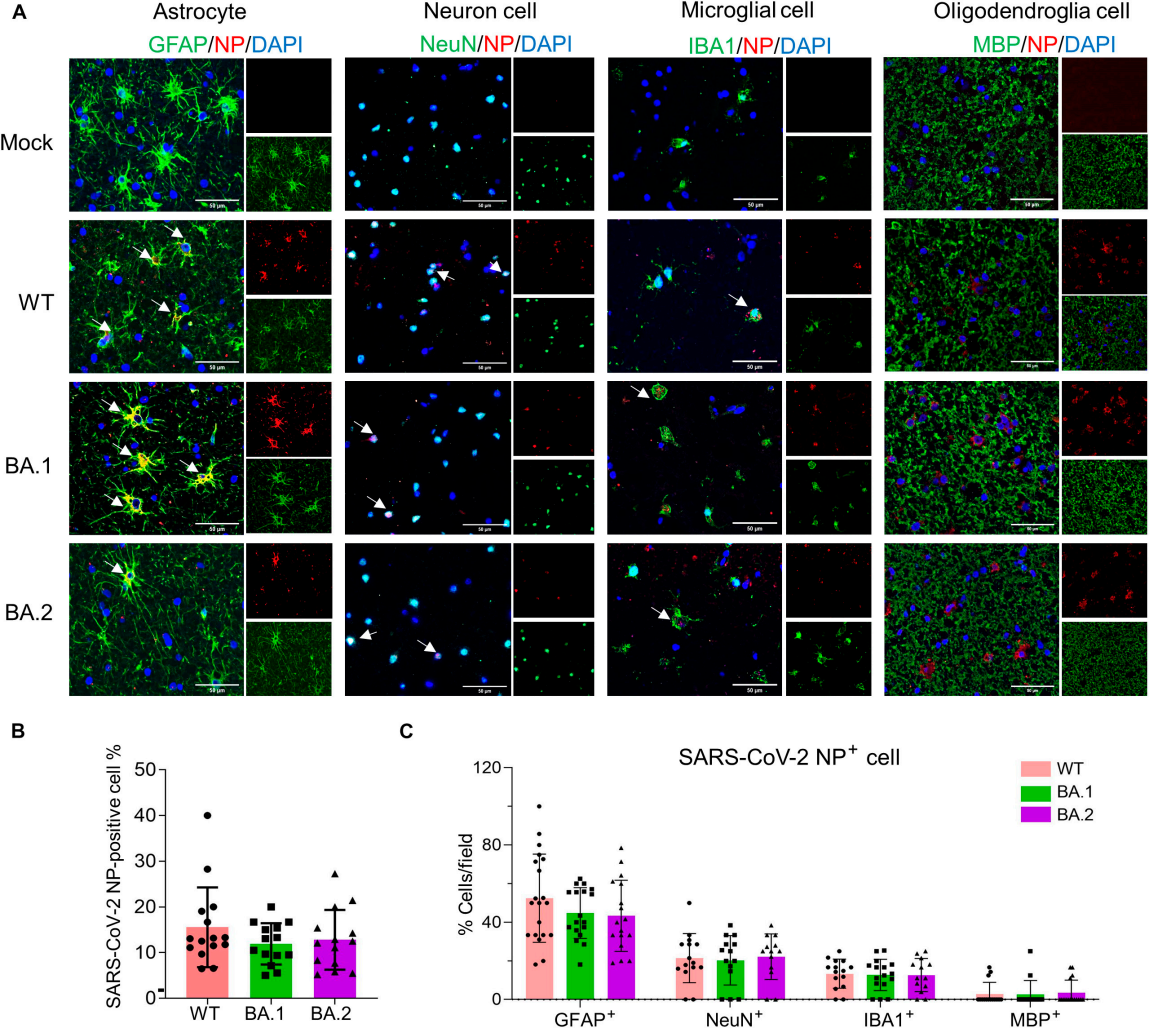
Cellular tropism of WT and Omicron variants in elderly BSs. (A) IFA staining of WT-, BA.1-, and BA.2-infected astrocytes, neurons, microglia, and oligodendrocytes in BSs at 72 hpi. Viral NP protein was stained red. Nuclei are shown with DAPI in blue. Scale bars, 50 μm. (B) Percentage of NP-positive cells in the spheroids from the 4 individual samples. Three to 5 fields per sample were analyzed. (C) Percentage of GFAP^+^, NeuN^+^, IBA1^+^, and MBP^+^ cells among all infected cells in the spheroids from the 4 individual elderly brain samples (3 temporal lobes and 1 frontal lobe). Three to 5 fields per sample were analyzed. Data represent the means ± SD; statistical significance was determined with one-way ANOVA (B and C).

The predictability of SARS-CoV-2 virulence evolution is complex because of various mechanisms, such as changes in transmission routes and tropism, competition within the host, and interactions with the immune system [25,26]. To identify whether the Omicron variant has different neurotropisms contribute to their less brain damage compared with WT strains, we utilized markers of astrocytes, neurons, microglia, oligodendrocytes, and endothelial cells costained with viral NP protein in BSs derived from 4 elderly individual brain samples (3 temporal lobes and 1 frontal lobe). On average, NP protein was detected in 15.55%, 11.92%, and 12.82% of the cells in WT-, BA.1-, and BA.2-infected BSs, respectively (Fig. 4A and B). However, the difference in infection levels did not reach statistical significance. We observed widespread infection in astrocytes regardless of the SARS-CoV-2 strain, with approximately 50% of infected cells expressing glial fibrillary acidic protein (GFAP) (Fig. 4A and C). However, there was no significant difference in infections of astrocytes, neurons, microglia, and oligodendrocytes between the WT and Omicron BA.1/BA.2 strains (Fig. 4A and C). We also found that brain endothelial cells can be infected with WT and Omicron BA.1/BA.2 (Fig. S3). These findings suggest that SARS-CoV-2 tends to infect astrocytes, consistent with previous human postmortem brain study [27], but Omicron BA.1/BA.2 does not show a difference in neurotropism compared to the WT strain in the aging brain.

### SARS-CoV-2 Omicron BA.1/BA.2 infection induces a weaker neuroinflammatory response than the WT strain in BSs

**Fig. 5.**
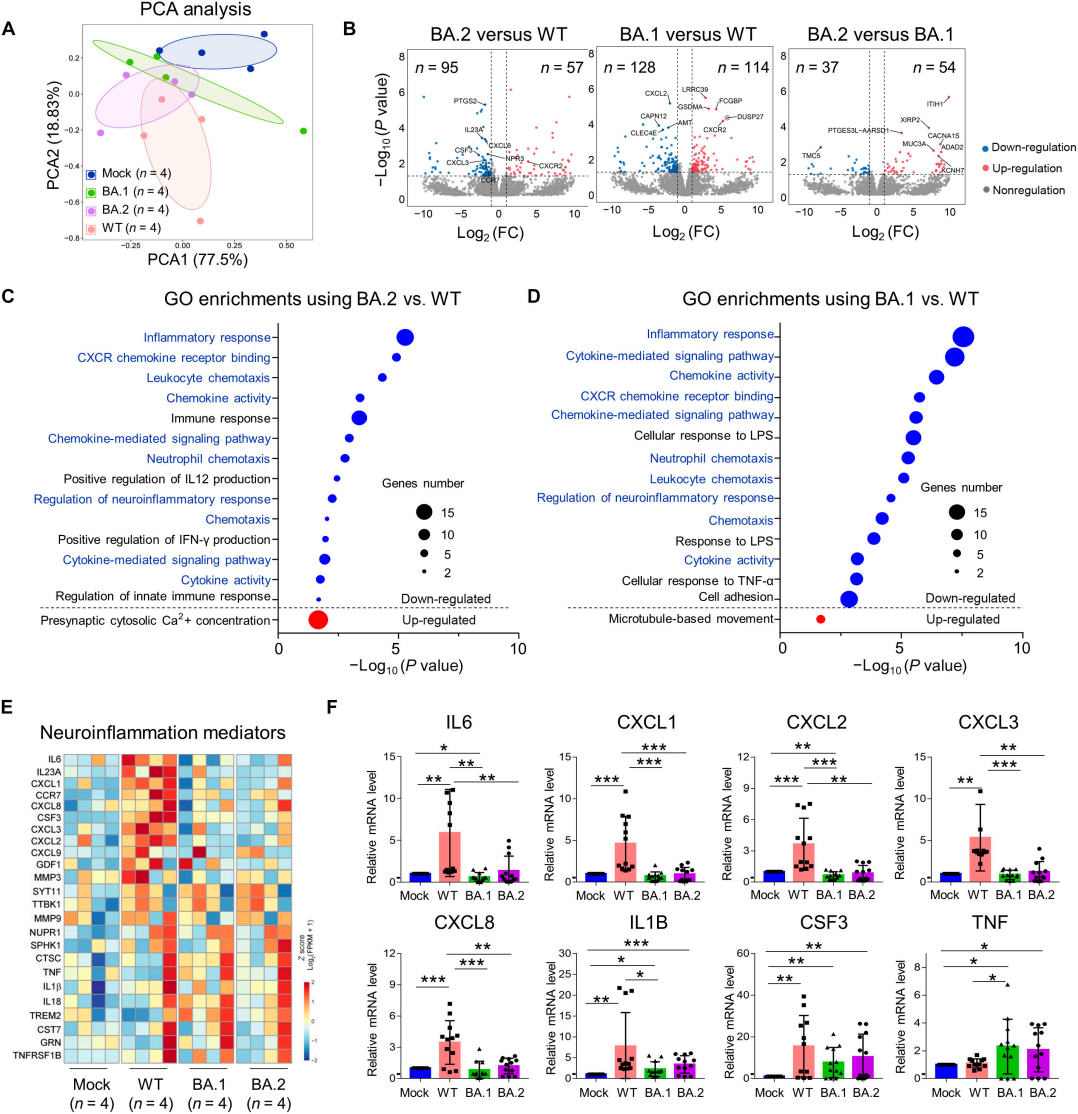
Omicron BA.1/BA.2 down-regulates the inflammatory response compared to the WT strain. (A) PCA was performed for SARS-CoV-2-infected and mock-infected BSs from 4 individual elderly brain samples (3 temporal lobes and 1 frontal lobe) at 72 hpi. (B) Volcano plots of differentially expressed genes in WT and BA.1/BA.2 compared with each other in BSs. FC, fold change. (C and D) GO analysis of up-regulated and down-regulated genes when comparing BA.1/BA.2 with the WT strain in BSs at 72 hpi. IFN-γ, interferon-γ; LPS, lipopolysaccharide. (E) Heatmap of neuroinflammatory mediator genes when comparing each SARS-CoV-2 strain with mock-infected BSs. FPKM, fragments per kilobase of transcript per million fragments mapped. (F) Inflammation-related gene (*IL6, CXCL1, etc.*) mRNA levels in SARS-CoV-2- and mock-infected BSs from the 4 individual samples, as assessed by reverse transcription qPCR with 3 technical replicates. Data represent the means ± SD; statistical significance was determined with one-way ANOVA. *P < 0.05, **P < 0.01, and ***P < 0.001.

To uncover mechanisms of SARS-CoV2 Omicron-induced ameliorated neuropathological injury, we performed RNA-seq analysis in control, WT-, and Omicron BA.1/BA.2-infected BSs derived from 4 elderly individual brain samples (3 temporal lobes and 1 frontal lobe) at 72 hpi. Principal components analysis (PCA) showed clustering of biological replicates within different SARS-CoV-2 strain infection groups (Fig. 5A). Interestingly, comparing Omicron variants with the WT strain, a volcano plot showed that many chemokines/cytokines, including CXCL3, CXCL2, and IL23A, were down-regulated in BA.1/BA.2-infected BSs (Fig. 5B). Comparing BA.2 or BA.1 with the WT strain, Gene Ontology (GO) and Kyoto Encyclopedia of Genes and Genomes (KEGG) analysis highlighted the common down-regulated pathways related to involve in immune response and inflammatory response (Fig. 5C and D and Fig. S4). Closer examination of gene sets involved in neuroinflammation mediators found that the WT strain induced up-regulation of a broader range of inflammation-related genes compared with the BA.1 or BA.2 strain (Fig. 5E). We confirmed reduced induction of a subset of genes (*IL6, CXCL1, CXCL2, CXCL3, CXCL8, *and* IL1B*) by Omicron BA.1 or BA.2 compared with WT and validated an increase in colony-stimulating factor 3 (*CSF3*) and tumor necrosis factor (*TNF*) after infection with all evaluated SARS-CoV-2 strains by quantitative polymerase chain reaction (qPCR) (Fig. 5F). Altogether, the evidence indicates that SARS-CoV-2 infection triggers neuroinflammation, while Omicron BA.1/BA.2 infection elicits a less neuroinflammatory response compared to the original strain in elderly BSs. This may explain why SARS-CoV-2 Omicron BA.1 or BA.2 is less neuropathogenic than the WT strain.

### SARS-CoV-2 Omicron BA.1/BA.2 and WT strain infections disrupt neural network activity associated with neurodegenerative disorders

**Fig. 6.**
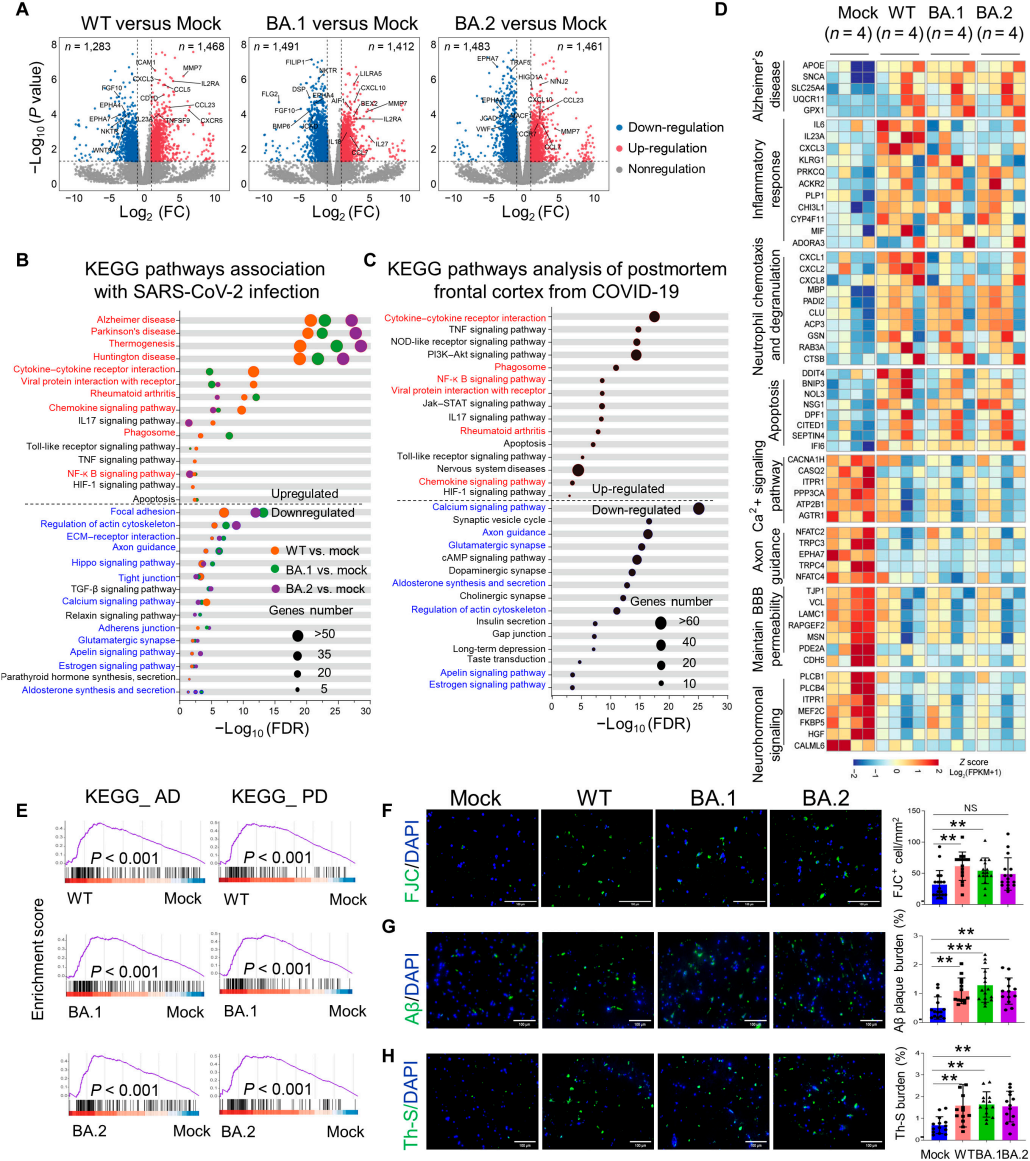
Omicron BA.1/BA.2 and WT infections are associated with neurodegenerative disorders. (A) Volcano plots of differentially expressed genes in SARS-CoV-2-infected compared with mock-infected BSs at 72 hpi. (B) KEGG analysis for up-regulated and down-regulated genes when comparing SARS-CoV-2 with mock-infected BSs. The common regulated pathways are labeled with the same color (down-regulated terms, blue; up-regulated term, red). NF-κB, nuclear factor κB; HIF-1, hypoxia-inducible factor 1; ECM, extracellular matrix; FDR, false discovery rate. (C) KEGG analysis for up-regulated and down-regulated genes comparing COVID-19 with control frontal cortex differentially expressed genes. NOD, nonobese diabetic; PI3K, phosphatidylinositol 3-kinase; Jak, Janus kinase; STAT, signal transducers and activators of transcription; cAMP, adenosine 3′,5′-monophosphate. (D) Heatmap of key differentially expressed genes when comparing each SARS-CoV-2 strain with mock-infected BSs. BBB, blood–brain barrier. (E) Gene set enrichment analysis of AD- and PD- related differentially expressed genes in SARS-CoV-2-infected BSs compared with the mock group. (F to H) IFA analysis with Fluro-Jade C (FJC) (F), Aβ (G), and thioflavin-S (H) in SARS-CoV-2-infected elderly BSs. Nuclei are shown with DAPI in blue. Scale bars, 100 μm. Quantification of positive cell per area (F to H) in BSs from 4 individual elderly brain samples (3 temporal lobes and 1 frontal lobe). Three to 5 fields per sample were analyzed. Data represent the means ± SD; statistical significance was determined with one-way ANOVA. **P < 0.01 and ***P < 0.001. NS, not significant (*P* > 0.05).

To further assess the functional consequences of infection with different SARS-CoV-2 strains in BSs at the molecular level, we reanalyzed the preceding RNA-seq dataset. Thousands of genes were differentially expressed in WT- and Omicron BA.1/BA.2-infected BSs compared with uninfected controls at 72 hpi (Fig. 6A). KEGG and GO analyses of up-regulated genes at 72 hpi revealed common enrichment for genes associated with neurodegenerative disease, viral transcription, immune response, cytokine- and chemokine-mediated signaling pathways, neutrophil chemotaxis and degranulation, and cell apoptosis for both WT and Omicron BA.1/BA.2 strains (Fig. 6B and D and Fig. S5A). Conversely, KEGG and GO analyses of down-regulated genes showed common enrichment for genes related to cytoskeleton remodeling, calcium signaling pathway, axon guidance, neurohormonal signaling pathways, and maintaining blood–brain barrier permeability for both strains (Fig. 6B and D and Fig. S5A). We further validated that several pathway networks in WT- and Omicron-infected BSs were similar to those in brain autopsy tissues of patients with COVID-19 (Fig. 6C and Fig. S5B) [28]. Taken together, SARS-CoV-2 WT and Omicron activate broad immune response and disrupted neural network activity in elderly BSs.

There is growing evidence to suggest that COVID-19 infection may disrupt the progression of Parkinson’s disease (PD) and Alzheimer’s disease (AD), potentially worsening symptoms or accelerating disease progression [29,30]. We aimed to directly investigate the potential link between COVID-19 and neurodegenerative disorders. First, we found strong associations between neurodegenerative diseases, including AD and PD, and WT and Omicron BA.1/BA.2 infections (Fig. 6E). In addition, we observed the presence of Fluoro-Jade C-positive degenerating neurons in WT- and Omicron BA.1/BA.2-infected BSs, which were not present in mock-infected spheroids or their original brain tissue (Fig. S6A), with no significant differences in the numbers of degenerating neurons between the 2 strains (Fig. 6F). We also focused on the preclinical marker of AD, cellular Aβ, and found that cellular Aβ aggregates were present in WT- and Omicron BA.1/BA.2-infected spheroids but not in mock-infected spheroids or their original brain tissue (Fig. 6G and Fig. S6B). Immunofluorescence analysis with thioflavin-S confirmed that the cytoplasmic deposition of Aβ in WT- and Omicron BA.1/BA.2-infected spheroids consisted of aggregated Aβ, which was not observed in their original brain tissue (Fig. 6H and Fig. S6C). Therefore, infection with WT and Omicron BA.1/BA.2 strains may potentially trigger or worsen neurodegenerative disorders in older individuals.

## Discussion

In this study, we provided a rapid and efficient way of generating 3-dimensional elderly BSs from surgically removed elderly human brain tissue by avoiding single-cell dissociation. These BSs recapitulate, at least partially, the multicellular composition and native cell–cell interactions of the parent brain tissue. This is supported by histology and immunohistology showing similar tissue organization and various cell types, as well as snRNA-seq demonstrating the preservation of different cell populations and their gene expression patterns. Recently, human fetal brain organoids with consistent molecular profiles and cellular heterogeneity were established using a similar methodology [31]. Because of reduced adult neurogenesis, the proportion and subpopulation of neurons change over culture time in spheroid culture systems, which needs further optimization in the future.

We further utilized elderly BSs to study different SARS-CoV-2 strain infections and neuropathological injuries. Our data showed that elderly BSs were permissive to WT and Omicron strain infections, but both WT and Omicron BA.1/BA.2 do not actively replicate in BSs Furthermore, we found that Omicron BA.1/BA.2 produced less infectious virus particles compared with WT. Previous study found that both SARS-CoV-2 WT and Omicron strains actively replicate in stem-cell-derived brain organoids [23,32]. The reason for this discrepancy may be due to stem-cell-derived brain organoids highly expressed ACE2 and endosomal entry proteases (cathepsin B and cathepsin L). However, ACE2 and TMPRSS2 actually lowly express, but alternative receptors such as NRP1 highly express in human brain tissue and our BSs model. Thus, the SARS-CoV-2-infected BSs model could reflect the natural infection conditions of brain tissue compared with stem cell models. More recently, some studies have shown that Omicron has a lower replication efficiency in the lungs and intestines than the Delta and WT strains [14,15,33]. The decreased infection of Omicron BA.1/BA.2 might be linked to newly acquired molecular changes such as extensive mutations in the receptor binding domain of the spike protein that modulates cell entry [34,35], as well as mutations in nonstructural proteins (NSPs) that could potentially alter replication kinetics [36]. Recent studies using chimeric viruses bearing different spike proteins showed that spike is a major determinant of the Omicron-attenuated replicative phenotype [26,37,38]. In addition, a study demonstrated that Omicron NSP6 contains critical attenuating mutations, which weakened the movement of lipids to replication organelles and decreased the replication of viral RNA [39]. SARS-CoV-2 open reading frame 3a and spike proteins down-regulate tetherin, antiviral restriction factor, and promotes the release of virus [40]. It is likely that these differences in the spike protein and NSPs in Omicron BA.1/BA.2 influence their replication in the brain.

On the other hand, we found that Omicron BA.1/BA.2 was less neuropathogenic than the WT strain. Recent studies have revealed the markedly attenuated pathogenicity of Omicron in mice, hamsters, and rhesus macaques compared to the Delta and WT strains [15,41,42]. Although the attenuated pathogenicity of Omicron has been widely confirmed, the mechanisms remain unclear. The Omicron variant is a markedly different variant with numerous mutations, particularly in the spike protein and NSPs [43–45]. Some of these mutations are of concern and may be associated with their reduced pathogenicity. A study demonstrated that mutating NSP6 along with the spike protein was sufficient to recapitulate the weakened characteristics of Omicron, indicating that both the spike protein and NSP6 play crucial roles in determining the attenuated phenotype of Omicron BA.1 [46]. Thus, Omicron may have reduced clinical severity, including neurological symptoms.

We assessed the difference in within-host interactions due to Omicron variant infection using the transcriptome. In contrast to WT, Omicron BA.1/BA.2-infected elderly BSs showed a reduced neuroinflammatory response. Numerous proinflammatory cytokines exhibited decreased expression in Omicron-infected BSs compared to those infected with the WT virus, including IL6, CXCL1, CXCL2, and IL1B, which play a crucial role in attracting immune cells to infection sites. This finding is in concordance with recent in vivo inflammatory assessments in the olfactory bulb of Omicron-infected hamsters [47]. Microglia, as integral components of the immune system, serve as the brain’s sentinel cells and detectors of invading pathogens. The proliferation, accumulation, and activation of microglia are typical characteristics of brain inflammation. Excessive and prolonged neuroinflammatory responses can affect brain function, including synaptic dysfunction, inhibition of neurogenesis, neural damage, and cognitive impairment [48,49]. Therefore, the differences in the neuroinflammatory response induced by Omicron infection may be linked to the degree of its neuropathogenicity. However, the mechanism of Omicron-induced neuroinflammation reduction remains unclear. The latest finding revealed that the SARS-CoV-2 spike and envelope proteins can cause neuroinflammation through Toll-like receptor signaling without the need for viral infection by functioning as a pathogen-associated molecular pattern [50–52]. The mutations of Omicron spike and envelope proteins could be a factor in their decreased inflammatory responses. Postmortem evidence showed the existence of encephalitis and/or meningoencephalitis in patients with COVID-19 in the SARS-CoV-2 era [53,54]. Our research suggests that the risk of Omicron-induced encephalitis may be lower.

We further examined interaction networks among various SARS-CoV-2 strains and host based on BSs. The transcriptional dysregulation in BSs resulting from WT or Omicron infection indicated an inflammatory response and perturbed neural cell function. Notably, WT or Omicron infection is associated with neurodegenerative disorders, such as AD and PD. Patients with COVID-19 and survivors may suffer from a neurodegenerative syndrome, which includes symptoms such as memory loss, delirium, and cognitive deficits [55,56]. This suggests that there may be a cellular mechanism underlying these effects. We observed that Fluro-Jade C-labeled degenerating neurons and Aβ deposition were present in elderly BSs infected by WT and Omicron. A previous investigation demonstrated a shift in the distribution of tau protein from axons to the cell body, as well as increased tau phosphorylation in neurons of brain organoids following exposure to SARS-CoV-2 [16]. In addition, an unpublished study indicated that SARS-CoV-2 infects the cortexes of patients with COVID-19, leading to the induction of AD-like characteristics by activating cellular and molecular pathways associated with AD. Consequently, these findings revealed the importance of understanding the impact of different SARS-CoV-2 variants on neurodegenerative processes and underscore the need for further research in this area to elucidate the underlying mechanisms and potential therapeutic targets.

Our research has some limitations. First, the sample sizes of paracancerous tissues of brain tumors were extremely limited. The BSs derived from brain tissue of different individuals encompass a diverse range of cell types, but there is great heterogeneity in different spheroids, which may limit the interpretation of the results from SARS-CoV-2 infection. Second, the absence of examination of brain tissue from patients with COVID-19 confirmed the presence of Omicron in brain tissue and its contribution to neurological disorders. Further investigations involving patients with COVID-19 could offer additional insights into how Omicron variants induce neurological manifestations.

In summary, we observed attenuated neuropathological injury caused by Omicron BA.1/BA.2 compared to the WT strain based on BSs derived from elderly human brain tissue. The Omicron BA.1/BA.2 infection induced weaker neuroinflammation than the WT strain, which may explain its neuropathological changes. In addition, both WT and Omicron infections triggered cellular and molecular neurodegenerative phenotypes. These findings provide a scientific basis for public health authorities to address the effect of COVID-19 physical, psychological, and functional sequelae to enhance the health and well-being of elderly individuals during the worldwide Omicron pandemic.

## Materials and Methods

### Human tissue sample collection

BSs were derived from adjacent tissues in patients with non-COVID-19 primary brain tumor who underwent surgical resection. A total of 9 patients were included in the present study. Table S1 summarizes the basic patient information for each subject. The collection and use of human brain samples in our research were approved by the Ethics Committee of Sun Yat-sen University Cancer Center (approval no. B2020-165-01). Written informed consent was provided by the study participants.

### Viruses and biosafety

WT SARS-CoV-2 (isolate Guangdong/20SF014/2020; EPI_ISL_403934) and the Omicron BA.1 and Omicron BA.2 strains were obtained from Guangdong Provincial Center for Diseases Control and Prevention and handled in a BSL-3 laboratory. The genome sequences of the BA.1 and BA.2 strains used in this study have been deposited in the GenBase of National Genomics Data Center (https://ngdc.cncb.ac.cn/) with submission numbers C_AA011261.1 and C_AA011262.1.

### SARS-CoV-2 infection

Cultured BSs (1 to 2 weeks) were infected in a 6-well ultralow attachment plate at an MOI of 0.1 in spheroid culture medium. After virus exposure for 4 to 6 h, spheroids were washed 3 times with advanced F12 and returned to culture with spheroid culture medium. Supernatant and spheroid samples were collected at designated time points after inoculation.

### Quantification and statistical analysis

All measurements are shown as the means ± SDs where appropriate. One-way analysis of variance (ANOVA) was used for comparison of the difference between mock-, WT- and Omicron-variant-infected BSs. Cells infected with SARS-CoV-2 were manually counted over total 4′,6-diamidino-2-phenylindole-positive (DAPI^+^) cells using the Cell Counter of ImageJ (National Institutes of Health). Statistical analysis was performed using GraphPad Prism 9. *P* < 0.05 was considered statistically significant. *P < 0.05, **P < 0.01, and ***P < 0.001; NS indicates not significant (*P* > 0.05).

Additional methods are provided in the Supplementary Materials.

## Data Availability

All data are available in the main text or the Supplementary Materials.
