# Supplementary material for: SARS-CoV-2 Omicron Variants Show Attenuated Neurovirulence Compared with the Wild-Type Strain in Elderly Human Brain Spheroids
Source: Research (Wash D C). 2024 May 13;7:0376. doi: 10.34133/research.0376 (PMC11089278; doi:10.34133/research.0376)
Supplement: Supplementary 1 — Materials and Methods Figs. S1 to S6 Tables S1 and S2 [file research.0376.f1.docx]

Supplementary Materials for

**SARS-CoV-2 Omicron variants show attenuated neurovirulence compared with the wild-type strain in elderly human brain spheroids**

**This PDF file includes:**

Materials and Methods

**Fig S1.** BSs retain cellular diversity of parental brain tissue

**Fig S2.** The replication level of SARS-CoV-2 WT and Omicron BA.1/BA.2 in elderly brain spheroids.

**Fig S3.** Brain endothelial cells were infected with SARS-CoV-2 WT and Omicron BA.1/BA.2 in elderly brain spheroids.

**Fig S4.** Inflammatory pathways analysis of SARS-CoV-2 different strains infected brain spheroids.

**Fig S5.** Transcriptome analysis of SARS-CoV-2-infected brain spheroids and postmortem brain tissue from COVID-19 patients.

**Fig S6.** Parental brain tissue not show neurodegenerative phenotype.

Table S1. Clinical information of brain tissue samples included in the study

Table S2. Primers used for reverse-transcription real-time quantitative PCR

**Materials and methods**

Antibodies and reagents

Antibodies used include the following: mouse anti-SARS-CoV-2-NP (Sino Biological, 40143-MM05, 1:100), rabbit anti-SARS-CoV-2-NP (Novus, NB100-56576, 1:100), rabbit anti-GFAP (Proteintech, 16825-1-AP, 1:200), rat anti-GFAP (Invitrogen, 13-0300, 1:200), rabbit anti-IBA1 (Proteintech, 10904-1-AP, 1:200), rabbit anti-MBP (Proteintech, 10458-1-AP, 1:200), rabbit anti-NeuN (Proteintech, 26975-1-AP, 1:200), rabbit anti-CD31 (Cell Signaling Technology, 77699S, 1:500), rabbit anti-Ki67 (Novus, NB500-170, 1:500), rabbit anti-ACE2 (Sino Biological, 10108-T24, 1:200), rabbit anti-TMPRSS2 (Proteintech, 14437-1-AP, 1:200), mouse anti-CD147/BSG (Proteintech, 66443-1-Ig, 1:200), anti-NRP1 (Proteintech, 60067-1-Ig, 1:200), rabbit anti-ZO-1 (Proteintech, 21773-1-AP, 1:200), rabbit anti- Beta Amyloid (Proteintech, 25524-1-AP, 1:200).

The culture of human brain spheroids

The adjacent normal tissues from patients were minced into approximately 1 mm diameter pieces using fine dissection scissors (LIGE, China) in Hibernate A medium (BrainBits) with 1x GlutaMax (Thermo Fisher Scientific) and 1x Antibiotic-antimycotic (Thermo Fisher Scientific). Tissue pieces were washed with DMEM:F12 medium to remove cellular debris and distributed in ultra-low attachment 6-well culture plates (Corning) with 4 mL of brain spheroids medium containing 50% DMEM:F12 (Thermo Fisher Scientific), 50% Neurobasal (Thermo Fisher Scientific), 1x GlutaMax (Thermo Fisher Scientific), 1x NEAAs (Thermo Fisher Scientific), 1x PenStrep (Thermo Fisher Scientific), 1x N2 supplement (Thermo Fisher Scientific), 1x B27 with vitamin A supplement (Thermo Fisher Scientific), 1x 2-mercaptoethanol (Procell, China), 2 ng/ml BDNF (MCE, HY-P7116A) and 2.5 μg/ml human insulin (Procell, China) per well and placed on an orbital shaker rotating at 120 rpm within a 37˚C, 5% CO2, and 90% humidity sterile incubator. Roughly 75% of the medium was changed every 48 hours by tilting the plates at a 45 angle and aspirating the medium above the sunken brain spheroids. The criteria for successful establishment of brain spheroids from a given patient's tissue was that the micro-dissected tissue pieces survived for 2 weeks, having a smooth surface.

Immunofluorescence and immunohistochemistry

Brain spheroids were fixed with 4% paraformaldehyde overnight after 72 hours post-infection. Spheroids were embedded in 3% low melting point agarose (Sigma-Aldrich), then embedded in paraffin. The tissue sections were cut to 8 μm thickness onto frosted glass slides. For deparaffinization, slides were baked at 60℃ for 1 h, followed by rehydration in xylene substitute (Cojet, China), and descending concentrations of ethanol (xylene substitute twice for 10 min; 100%, 90%, 80%, 70% ethanol, 1x PBS once; each step for 5 min). Heat-induced epitope retrieval was performed in a pressure cooker using Citrate Antigen Retrieval Solution (pH=6, ECOTOP, China) at 120℃ for 2 min and cooled down to room temperature. Slides were washed for 5 min in PBS with 0.1% Tween 20 (PBST). Sections were then blocked in 10% normal goat serum (NGS, Solarbio, China) in PBST at room temperature for 1 h, followed by incubation with primary antibodies in 1% NGS. After overnight incubation of primary antibodies at 4℃, sections were washed three times with PBST and stained with the appropriate secondary antibodies in PBS at room temperature for 1.5 h. Following this, sections were washed and stained with DAPI (Solarbio, China). After washed with PBST, sections were mounted with Mounting Medium (ZSGB, China).

Images were acquired using confocal microscope (OLYMPUS, FV3000) and TissueFAXS PLUS system (TissueGnostics). The number of cells was counted on DAPI+ nuclei from 3-5 pictures of representative areas in brain spheroids from four individual samples by visual scoring of color micrographs of lesions, and positive cells for every single marker and markers combination were calculated.

Multiplex immunohistochemistry (mIHC)

mIHC was performed using a Branch TSA 4-color combination kit (TissueGnostics) according to the manufacturer’s instructions. In brief, slides were deparaffinized and dehydrated the same steps as above. Sections were then cross-linked to slides by incubation in 10% formalin for 10 min. After two 5-min washes in distilled water, antigen retrieval in EDTA Antigen Retrieval solution (pH=8, Solarbio, China) was performed in a microwave. For each mIHC cycle, sections were blocked with antibody blocking solution for 10 min before incubation for 30 min at 37℃ with the appropriate primary antibody after cooling on the ice for 20 min. Then, slides were washed in 1x Tris-buffered saline with Tween-20 buffer three times and incubated with pre-diluted horseradish peroxidase (HRP)-conjugated secondary antibody for 10 min at room temperature. Next, sections were incubated in tyramide-fluorophore conjugate and amplification reagent (TissueGnostics) for 10 min at room temperature. To elute the antibodies, slides were boiled in a microwave again. The cycle was repeated for reaction with the next antibody. The process was performed on the following antibodies and fluorescent dyes in the flowing order: NeuN/TG570N, IBA1/TG620N, MBP/TG700N, GFAP/TG520N, DAPI. Sections were scanned and imaged using the TissueFAXS PLUS system (TissueGnostics).

Single-nucleus RNA-seq on the 10x Genomics platform

Parental tissue and cultured spheroids were frozen until processing in the same nuclei isolation batch to minimize potential batch effects. Samples were homogenized in 2 ml of Nuclei EZ Lysis buffer (Sigma-Aldrich) supplemented with protease inhibitor (Roche) and RNase inhibitor (Promega) using a Dounce homogenizer (Kimble Chase) on ice. And they were incubated on ice for 5 minutes with an additional 2 ml of lysis buffer. The homogenate was filtered through a 40-mm cell strainer (pluriSelect) and then centrifuged at 500 g for 5 minutes at 4°C. The pellet was resuspended and washed with 4 ml of the buffer, and then, it was incubated on ice for 5 minutes. After another centrifugation, the pellet was resuspended in Nuclei Suspension Buffer (1x PBS,0.07% BSA, and 0.1% RNase inhibitor), filtered through a 20-mm cell strainer (pluriSelect), and counted. Libraries were generated and sequenced using the Chromium Next GEM Single Cell 3’ GEM, Library & Gel Bead Kit (v3) according to manufacturer protocols.

Single nucleus analysis

CellRanger v6.0 (https://github.com/10X Genomics/cellranger) preliminarily analyzed the single cell data (fastq format) and aligned it with the Homo sapiens GRCh38 reference genome. Low quality cells were next filtered with a median of < 500 or > 5000 genes, proportion of mitochondrial genes> 30%, and number of UMI <500. Then expression matrix files for each cell were generated (mtx or tsv formats). Further subsequent analysis of the single-cell expression matrix was performed by Seurat v4.0 (https://satijalab.org/seurat/). Dimension reduction clustering of cells using tSNE clustering algorithm to perform differential analysis for genes in different cluster. Through these analyses, single-cell subpopulation characteristics in different groups were compared.

RNA extraction and RT–qPCR

Spheroid samples were collected and extracted with the RNeasy Mini kit (Qiagen, 74106). Supernatant samples were extracted with the viral RNA Mini kit (Qiagen, 52906). SARS-CoV-2 replication was detected by RT-qPCR kit (Daan Gene, Guangzhou, China). To detect expression level of host genes, samples were performed with reverse transcription using the HiScript III 1st Strand cDNA synthesis Kit (Vazyme, China). cDNA was diluted 5 times and subjected to real-time PCR using ChamQ Universal SYBR qPCR Master Mix (Vazyme, China). Primer sequences are available in Table S2.

Transmission electron microscopy

SARS-CoV-2- and mock-infected BSs were fixed with 2% paraformaldehyde–2.5% glutaraldehyde solution at 4 °C overnight. Then, the spheroids were dehydrated in gradient ethanol and embedded in epoxy resin PON812 and polymerized at 60 °C for 24 h. Ultrathin sections (70 nm thickness) were obtained and transferred onto 200 Mesh copper grids covered with a formvar and carbon film. Sections were post-stained with uranyl acetate and lead citrate. All TEM data were observed under transmission electron microscope (FEI Tecanai G2 Spirit Twin, USA).

Viral titration by TCID50 assay

A confluent 96-well tissue culture plates of Vero-E6 cells was prepared one day before the virus titration (TCID50) assay. Cells were washed once with PBS and replenished with DMEM with 2% foetal bovine serum supplemented with PenStrep. Serial dilutions of virus supernatant, were performed and each virus dilution was added to the plates. The plates were observed for cytopathic effect daily. The end point of viral dilution leading to CPE in 50% of inoculated wells was estimated using the Karber method.

TUNEL assay

Apoptosis in paraffin-embedded tissue of SARS-CoV-2-infected and mock-infected BSs was detected by In Situ Cell Death Detection Kit-FITC (Roche) according to the manufacturer’s protocols. Briefly, slides were permeabilized with 0.1% Triton X-100 for 2 mins. After washing three times in TBS, TUNEL label and TUNEL enzyme were mixed in a proportion of 10:1 and added to slides for 1 hour at 37℃. Slides were washed and incubated with DAPI and analyzed using confocal microscope (OLYMPUS, FV3000).

Luxol Fast Blue staining

Luxol Fast Blue was used for the visualization of myelin sheath. Slides were deparaffinized and dehydrated the same steps as above. Then, slides were placed in 0.1% Luxol Fast Blue (Servicebio, China) preheated for 30 min and incubated for 4 h at 65℃. After cooling at room temperature, slides were washed with tap water until the water was colorless. The slides were quickly immersed in lithium carbonate (Servicebio, China) for 5 s, and then immediately placed in 70% ethanol for 10 s. The preceding steps were repeated for differentiation until the myelin sheath was blue with a nearly colorless background under microscopic examination, and then washed to terminate differentiation. The slides were dehydrated with 100% ethanol three times, 5 min each time, and then mounted with neutral balsam. The severity of white matter lesions was classified into four grades as described by Wakita et al. [1]: normal (grade 0), disarrangement of nerve fibers (grade 1), formation of marked vacuoles (grade 2), and disappearance of myelinated fibers (grade 3).

Bulk RNA sequencing

Sixteen total RNA samples were extracted from SARS-CoV-2 WT, Omicron BA.1/BA.2-infected and mock-infected BSs using RNeasy Mini Kit (Qiagen, Germany). RNA concentration and quality were assessed using a Nanodrop 2000 (Thermo Fisher Scientific). mRNA Purification Kit (Invitrogen) was applied to deplete rRNA and isolate poly(A)+ RNA by using oligo d(T). The isolated mRNA was fragmented using fragmentation reagents (Invitrogen). The short fragments were primed with random hexamers for first-strand cDNA synthesis, then second-strand cDNA synthesis. Next, adenine (A) nucleotide was added to the 3’ ends of the blunt fragments. During adapter ligation and amplification, indexes and adapters were added to both ends of the fragments. The libraries were sequenced on an Illumina Hiseq 4000 platform and 150-bp paired-end reads were generated.

Host RNA analysis of mRNA-seq

For host mRNA mapping, raw reads were aligned to human reference genome (version hg19/GRCh37). Differential gene expression analysis for BSs between Mock, 72 hpi SARS-CoV-2 infection of WT, BA.1 and BA.2 strains was performed using DESeq2 R package. P value < 0.05 and |log2 (fold change) | > 1 were set as threshold for significantly different expression. The DEGs were submitted to KOBAS 3.0 online (http://kobas.cbi.pku.edu.cn/) for GO enrichment and KEGG pathway analysis. From GO and KEGG pathway enrichment results, significant pathways (q value < 0.05) related to viral infection, immune system, neural signal transduction were selected for further investigation. The heatmap plot of gene expressions, the volcano plot of result of gene differential expression analysis, and the bar plot of pathways were generated using the R ggplot2 package. Venn diagrams were drawn by jvenn online (http://jvenn.toulouse.inra.fr/app/example.html).

Publicly available RNA-seq profiling of human frontal cortex in severe COVID-19 or unaffected patients were downloaded from the National Center for Biotechnology Information (NCBI) Gene Expression Omnibus website (GEO) with the accession numbers GSE188847. The Differential gene expression analysis of frontal cortex in severe COVID-19 compared with unaffected patients using DESeq2 R package. Then, GO enrichment and KEGG pathway analysis were performed as described above.

Quantification of viral gene expression levels using mRNA-seq data

Since SARS-CoV-2 transcript has a 5’-cap structure and a 3’ poly(A) tail, the short fragments of viral transcript can be enriched using Beads with oligo (dT). We collected all strains sequencing raw reads that are mapped to SARS-CoV-2/human/USA/WA-CDC-WA1/2020 genome (GenBank: MN985325.1). The leftmost mapped position in the reference was used as the junction site and the resultant positions were adjusted to the reference on the basis of a global alignment. Then, we calculated the number of reads in indicated SARS-COV-2 gene region (ORF1ab, S, ORF3a, E, M, ORF6, ORF7a, ORF7b, ORF8, N). Due to the mRNA-seq read lengths being long enough (100 nts) to be uniquely mapped, the numbers of the reads reflect the actual viral mRNA expression levels.

Fluro Jade C Staining

Fluro Jade C was used to stain degenerating neurons according to the manufacturer’s instructions (Solarbio, China). Slides were deparaffinized and dehydrated the same steps as above. After being dried naturally, slides were immersed in Pretreatment Solution A (1x) for 5 min, followed by 2 min in 70% ethanol and 2 min in distilled water. Slides were immersed in Pretreatment Solution B (1x) for bleaching for 10 min and then washed in distilled water for 2 min. Then, slides were incubated in prepared Working Solution C at temperature in the dark for 10 min. Followed by washing in distilled water three times for 1 min each time, sections were mounted with Mounting Medium (ZSGB, China).

Thioflavin-S staining

The sections were immersed in 0.5% thioflavin-S (MCE, HY-D0972) for 5 min, hydrated in 70% ethanol, and washed three times with PBS. After staining with Hoechst 33258 for 10 min, the sections were sealed with a fluorescent mounting media. Sections were scanned and imaged using the TissueFAXS PLUS system (TissueGnostics). The percentage of thioflavin-S-positive areas was determined to evaluate amyloid deposition.

Quantification and statistical analysis

All measurements are shown as the means ± SDs where appropriate. Paired *t* tests were used for comparison of viral gene copy number levels between WT and Omicron variant-infected BSs. Unpaired *t* tests were used to compare mock, WT and Omicron variant-infected cells. Cells infected with SARS-CoV-2 were manually counted over total DAPI+ cells using the Cell Counter of ImageJ (NIH). Statistical analysis was performed using GraphPad Prism 9. *p< 0.05* was considered statistically significant.

[1] Wakita H, Tomimoto H, Akiguchi I, Kimura J. Glial activation and white matter changes in the rat brain induced by chronic cerebral hypoperfusion: an immunohistochemical study. Acta Neuropathol. 1994;87(5):484-92.

Fig S1. BSs retain cellular diversity of parental brain tissue

**(A)** H&E staining images of parental brain tissue and corresponding spheroids. Scale bars, 200 μm. **(B)** Multiplex immunohistochemistry for neurons (NeuN), oligodendrocytes (MBP), astrocytes (GFAP), microglia (IBA1) markers in brain spheroids and their parental brain tissue. Scale bars, 50 μm. **(C)** Cell number of each sample used in snRNA-seq analysis. **(D)** Dot plot showing the average log-normalized expression of a set of marker genes and the fraction of cells expressing the genes in each unsupervised cluster. **(E)** UMAP plots of single-nuclei RNA expression in BSs after 2 or 4 weeks of culture. **(F)** Unsupervised clustering via UMAP of all cells pooled from parental tissue and corresponding BSs and annotated UMAP clusters of all cells. **(G)** UMAP plots of parental tissue and corresponding primary brain spheroids at 2 and 4 weeks colored by cell clusters, respectively. **(H)** The proportion of each cell cluster in parental tissue and corresponding primary brain spheroids at 2 and 4 weeks.

Fig S2. The replication level of SARS-CoV-2 WT and Omicron BA.1/BA.2 in elderly brain spheroids

**(A)** Brain spheroids were infected at MOI of 0.1, viral supernatant samples harvested at 72 hpi were quantified with RT-qPCR against SARS-CoV-2 NP gene. **(B)** The corresponding cell lysates were collected at 72 hpi. for quantifying NP gene of SARS-CoV-2. **(C)** Viral supernatant and cell lysate samples harvested at 72 hpi were quantified with RT‒qPCR against the NP gene. n=6 biological samples from different brain samples (5 temporal lobes, 1 frontal lobe; average age 64.8 years; range 57–72 years). **(D)** Immunofluorescence staining for SARS-CoV-2-related receptors in brain spheroids. Nuclei were shown with DAPI in blue. Scale bar: 50 μm. Data represent the mean ± SD from the indicated number of biological replicates. Statistical significance was determined with One-way ANOVA (C). **P<0.05*, ***P<0.01* and ****P<0.001*; *NS*, not significant (*P > 0.05*).

Fig S3. Brain endothelial cells were infected with SARS-CoV-2 WT and Omicron BA.1/BA.2 in elderly brain spheroids.

Immunofluorescence staining for SARS-CoV-2 NP protein (red) and CD31 (green) in brain spheroids. Nuclei were shown with DAPI in blue. Scale bar: 50 μm.

**Fig S4. Inflammatory pathways analysis of SARS-CoV-2 different strains infected brain spheroids.**

Dot plots of top KEGG enrichment analysis for upregulated and downregulated genes when comparing SARS-CoV-2 BA.1/BA.2 with WT strain in brain spheroids at 72 hpi.

**Fig S5. Transcriptome analysis of SARS-CoV-2-infected brain spheroids and postmortem brain tissue from COVID-19 patients. (A)** Dot plots of GO terms analysis for upregulated and downregulated genes when comparing SARS-CoV-2 WT, BA.1 and BA.2 with mock-infected brain spheroids at 72 hpi. **(B)** Dot plots of GO terms analysis for upregulated and downregulated genes when comparing COVID-19 with control frontal cortex. GO terms consistent with SARS-CoV-2-infected brain spheroids are shown in bold

**Fig S6. Parental brain tissue not show neurodegenerative phenotype.** Immunofluorescence analysis with Fluro-Jade C (**A**), Aβ (**B**) and thioflavin-S (**C**) in parental brain tissue. Nuclei were shown with DAPI in blue. Scale bar, 200 μm.

Table S1. Clinical information of brain tissue samples included in the study

| **Sample** | **Sex** | **Age** | **Diseases & Conditions** | **Harvest surgical site** |
| --- | --- | --- | --- | --- |
| 112251 | F | 66 | Glioblastoma | Temporal lobe |
| 112217 | F | 72 | Glioma | Temporal lobe |
| 112232 | M | 57 | Glioma | Temporal lobe |
| 112221 | M | 61 | Lung cancer with brain metastasis | Frontal lobe |
| 112213 | M | 65 | Colon cancer with brain metastasis | Temporal lobe |
| 112246 | F | 68 | Glioblastoma | Temporal lobe |
| 112368 | F | 59 | Glioblastoma | Frontal lobe |
| 112374 | M | 53 | Glioma | Frontal lobe |
| 112387 | F | 57 | Glioblastoma | Temporal lobe |

Table S2. Primers used for reverse-transcription real-time quantitative PCR

| **Gene** | **Forward** (5’ – 3’) | **Reverse** (5’ – 3’) |
| --- | --- | --- |
| IL6 | ACTCACCTCTTCAGAACGAATTG | CCATCTTTGGAAGGTTCAGGTTG |
| CXCL1 | AAGAACATCCAAAGTGTGAACG | CACTGTTCAGCATCTTTTCGAT |
| CXCL2 | AACCGAAGTCATAGCCACAC | CTTCTGGTCAGTTGGATTTGC |
| CXCL3 | CGCCCAAACCGAAGTCATAG | GCTCCCCTTGTTCAGTATCTTTT |
| CXCL8 | AACTGAGAGTGATTGAGAGTGG | ATGAATTCTCAGCCCTCTTCAA |
| IL1B | ATGATGGCTTATTACAGTGGCAA | GTCGGAGATTCGTAGCTGGA |
| CSF3 | GCTGCTTGAGCCAACTCCATA | GAACGCGGTACGACACCTC |
| TNF | CCTCTCTCTAATCAGCCCTCTG | GAGGACCTGGGAGTAGATGAG |
| GAPDH | GTCTCCTCTGACTTCAACAGCG | ACCACCCTGTTGCTGTAGCCAA |
